# Surgical outcomes and functional morbidity after retrosigmoid/retrocondylar and midline suboccipital approaches for foramen magnum meningiomas: a single-arm meta-analysis

**DOI:** 10.1007/s10143-026-04567-5

**Published:** 2026-11-09

**Authors:** Khushal Gupta, David Altschul, Michael Karsy, Vijay Agarwal

**Affiliations:** 1 The Leo M. Davidoff Department of Neurological Surgery, Albert Einstein College of Medicine, 1300 Morris Park Avenue, New York, NY 10461 USA; 2 Department of Neurosurgery, University of Michigan Medicine, Ann Arbor, MI USA

**Keywords:** Cranial nerve morbidity, Foramen magnum meningioma, Gross total resection, Midline suboccipital approach, Retrosigmoid approach, Systematic review

## Abstract

Foramen magnum meningiomas (FMMs) are surgically challenging due to their proximity to the cervicomedullary junction, lower cranial nerves, and vertebrobasilar circulation. Although far-lateral transcondylar approaches are commonly used for ventral lesions, less extensive posterior/posterolateral corridors are increasingly employed. We performed a single-arm meta-analysis to quantify oncologic control and functional morbidity following retrosigmoid/retrocondylar and midline suboccipital approaches for FMMs. A PRISMA-guided systematic review was conducted using PubMed/MEDLINE, Embase, Scopus, and Web of Science from inception through July 2025 (last search 31 July 2025). Eligible studies were original clinical case series with ≥ 5 patients (all eligible series proved retrospective) reporting FMM resection via posterior midline suboccipital or lateral/posterolateral suboccipital corridors, including retrosigmoid/retrocondylar approaches with limited condyle drilling ≤ 1/3 when performed. Studies with nonseparable outcomes from more extensive transcondylar/far-lateral approaches were excluded. Pooled proportions were calculated using a random-effects model with Freeman–Tukey transformation and reported with 95% confidence intervals. Seven retrospective case series from 1997 to 2019 contributing 133 eligible patients were included; one series was restricted to its 34 intradural cases operated through eligible corridors. Weighted mean age was 54.4 years, 75.2% (100/133) were female, and sample-size-weighted mean follow-up was 58 months. Retrosigmoid/retrocondylar corridors were used in 74/133 patients (55.6%) and midline suboccipital craniectomy in 59/133 (44.4%), often with C1 laminectomy. Dedicated retrosigmoid/retrocondylar series included predominantly anterior/anterolateral tumors, whereas midline series included mixed attachments. Formal approach-specific comparisons were limited by heterogeneous reporting. When performed, condyle drilling was generally limited to the posterior one-third; no postoperative craniocervical instability or fusion was reported. Pooled gross total resection was 91% (95% CI: 84–96%; 119/133 patients, 7 studies), recurrence was rare (3/133, 2.3%; pooled 1%, 95% CI: 0–4%), and long-term functional independence was 83% (95% CI: 71–93%; 60/74 patients, 5 studies). New cranial nerve deficits, transient or permanent, occurred in 21% (95% CI: 13–30%; 22/99 patients, 6 studies), cerebrospinal fluid leak in 12% (95% CI: 6–20%; 13/99, 6 studies), and perioperative mortality in 3% (95% CI: 0–9%; 7/133, 7 studies), with two additional late deaths from aspiration pneumonia during follow-up. Taken together, retrosigmoid/retrocondylar and midline suboccipital approaches for FMMs achieve high resection rates, low recurrence, and favorable long-term function, often without extensive condylectomy. These pooled estimates represent a combined figure across two anatomically distinct corridors applied to topographically different tumors (retrosigmoid/retrocondylar routes predominantly for anterior/anterolateral lesions and midline suboccipital exposure predominantly for posterior/posterolateral lesions), and do not imply that the approaches are interchangeable. Cranial nerve morbidity, cerebrospinal fluid leak, and perioperative mortality remain important considerations.

## Introduction

Foramen magnum meningiomas (FMMs) are uncommon skull base tumors arising at the craniocervical junction, where even modest growth can affect the cervicomedullary junction, lower cranial nerves (LCN IX–XII), and vertebrobasilar circulation [1–3]. FMMs are rare, accounting for approximately 1.5–3.2% of all intracranial meningiomas and 4–7% of meningiomas of the posterior fossa [1, 2]. Clinical presentation is often insidious and includes cervico-occipital headache, long-tract signs, gait disturbance, and variable bulbar dysfunction, reflecting the compact neurovascular anatomy of the region [3, 4].

Because ventral and ventrolateral FMMs lie anterior to the neuraxis, a direct posterior trajectory risks traction on the medulla and upper cervical cord; far-lateral and extreme-lateral transcondylar approaches were therefore developed to flatten the operative angle, provide early proximal control of the vertebral artery, and allow tumor devascularization before neural dissection. Historically, ventral and ventrolateral FMMs were consequently managed using such transcondylar variants to improve anterior brainstem access and reduce neural traction [2]. However, these approaches may require vertebral artery mobilization and condylar drilling, which can increase approach-related morbidity and, when extensive, raise concerns for craniocervical instability [5, 6]. Contemporary topographic and vertebral artery–based classification systems emphasize that approach selection should be dictated by tumor attachment, growth vector, and neurovascular displacement rather than a one-size-fits-all escalation to transcondylar exposure [1, 6].

Multiple single-center series suggest that many FMMs, including lesions with anterior/anterolateral components, can be safely resected through less disruptive posterior and posterolateral corridors [7–10]. Because this evidence is fragmented and heterogeneously reported, we performed a PRISMA-guided systematic review and single-arm meta-analysis to quantify tumor control and functional morbidity following retrosigmoid/retrocondylar and midline suboccipital approaches for FMMs [11].

## Materials & Methods

### Study design and search strategy

**Fig. 1:**
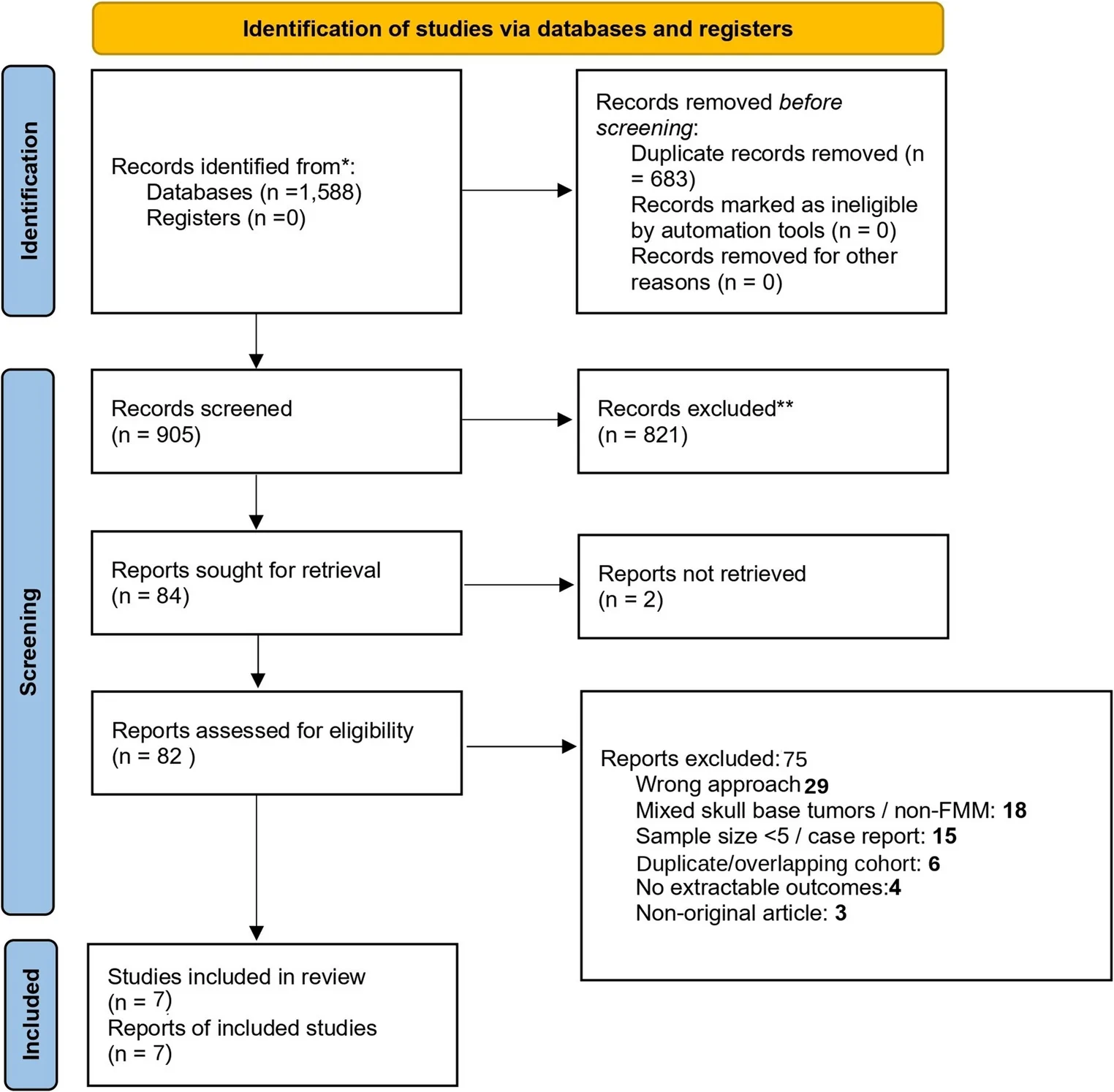
PRISMA diagram for studies in this systematic review. PRISMA 2020 flow diagram reproduced from Page et al. [11] Published under a Creative Commons Attribution (CC BY 4.0) license

We conducted a PRISMA-guided systematic review [11] and single-arm meta-analysis evaluating surgical outcomes and functional morbidity after retrosigmoid/retrocondylar and midline suboccipital approaches for FMMs (Fig. 1). A comprehensive literature search was performed in PubMed/MEDLINE, Embase, Scopus, and Web of Science databases. Search terms combined keywords and controlled vocabulary related to “foramen magnum,” “meningioma,” and operative corridors including retrosigmoid, retrocondylar, lateral/posterior suboccipital, and midline suboccipital. Reference lists of eligible studies were also screened to identify additional relevant reports. Search terms were combined using Boolean operators (AND/OR) with database-specific controlled vocabulary (MeSH for PubMed, Emtree for Embase) and free-text synonyms; no language or date restrictions were applied. The search was last run on 31 July 2025.

### Data extraction, study selection, and outcome measures

Studies were included if they (1) reported patients with radiographic and/or histopathologic diagnosis of FMM, (2) underwent resection via a posterior midline suboccipital corridor and/or a lateral/posterolateral suboccipital corridor (lateral suboccipital retrosigmoid/retrocondylar or “far-lateral retrocondylar”) with C1 laminectomy/laminoplasty permitted when described and with no or limited occipital condyle drilling (≤ 1/3), (3) were original clinical series with ≥ 5 patients, and (4) provided extractable outcome data for at least one endpoint of interest. No restriction to retrospective or single-center designs was applied; in practice, no prospective series meeting the remaining criteria were identified, and all included studies were retrospective. Case reports or small series (< 5), review articles/technical notes without outcomes, studies in which outcomes for the target approaches could not be separated from other skull base approaches, duplicate or overlapping cohorts (retaining the most complete report), and reports without extractable surgical outcome measures were excluded. Series primarily using transcondylar/extreme-lateral approaches with extensive condylectomy (> 1/3) and/or vertebral artery transposition were also excluded unless outcomes for the eligible corridors were reported separately. Where a mixed cohort provided patient-level data allowing individual patients to be assigned to eligible or ineligible corridors, only the eligible patients were retained; this applied to George et al. [3], from which the 34 intradural cases operated through posterolateral or midline posterior approaches were included and the 6 cases with an extradural component (treated through an anterolateral approach in 5, with vertebral artery transposition in 4 and more extensive drilling) were excluded. During revision, all 31 reports previously excluded at full text for approach-related reasons were re-screened against these criteria.

The methodological quality of included studies was assessed using the Joanna Briggs Institute (JBI) critical appraisal checklist for case series, a structured critical-appraisal tool appropriate for observational case series. Two reviewers independently evaluated each study across key domains including clarity of objectives, patient selection, ascertainment of diagnosis and outcomes, completeness and duration of follow-up, and reporting transparency. Each item was scored as 1 (criterion met) or 0 (not met/unclear). ‘Not applicable’ items were excluded from the denominator. A total quality score (sum of items) and percent score (total/eligible items ×100) were calculated for each study to facilitate visualization; a percent score of 100% indicates that all applicable criteria were met (highest methodological quality). Discrepancies were resolved by consensus.

We included original clinical surgical cohorts/case series reporting outcomes after resection of foramen magnum meningiomas (FMMs) performed via retrosigmoid/retrocondylar or midline/posterior suboccipital approaches. Data extraction was performed in duplicate using a standardized form. Extracted variables included study characteristics (year, design, cohort size), patient demographics, tumor features (including location/ventrality where described), surgical approach details (retrosigmoid/retrocondylar vs. midline suboccipital, extent of condylar drilling when reported), follow-up duration, use of adjuvant radiotherapy/radiosurgery, and clinical outcomes/complications. Discrepancies were resolved by consensus.

Primary outcomes were gross total resection (GTR) (as defined by each study) and long-term functional independence, defined as a good functional outcome on a validated scale at last follow-up (Glasgow Outcome Scale 4–5, Karnofsky Performance Status ≥ 80, or modified McCormick grade I–II); series reporting only global clinical improvement without such a scale were not pooled for this outcome. Secondary outcomes included new postoperative cranial nerve deficits (any new deficit, transient or permanent), cerebrospinal fluid (CSF) leak, tumor recurrence/progression, and perioperative mortality, defined as death within 30 days of surgery or during the index hospitalization as reported by each study; deaths occurring later during follow-up were recorded separately and are reported descriptively. Neurological improvement relative to the preoperative status was recorded by most series on the same scale as functional independence and could not be defined as a distinct outcome; it was therefore not pooled and is described narratively. For every pooled estimate, the number of contributing studies and patients is stated together with the crude event count.

### Statistical analysis

For quantitative synthesis, we performed a single-arm meta-analysis of proportions using a random-effects model. Proportions were stabilized with the Freeman–Tukey double-arcsine transformation, and pooled estimates were reported with 95% confidence intervals. Between-study heterogeneity was assessed using I². All quantitative syntheses (forest plots and pooled proportions) were conducted using Meta-Analysis Online (metaanalysisonline.com) [12]. Formal subgroup analyses, meta-regression, and sensitivity analyses were not performed given the limited number of eligible studies (k = 7), which precludes meaningful statistical exploration of heterogeneity sources. Funnel plot–based assessment of publication bias was similarly not conducted, as this method requires a minimum of approximately 10 studies to yield interpretable results. Formal certainty-of-evidence grading (GRADE) was not applied, as all included studies were retrospective case series representing inherently low-certainty evidence.

### Data sharing

**Fig. 2:**
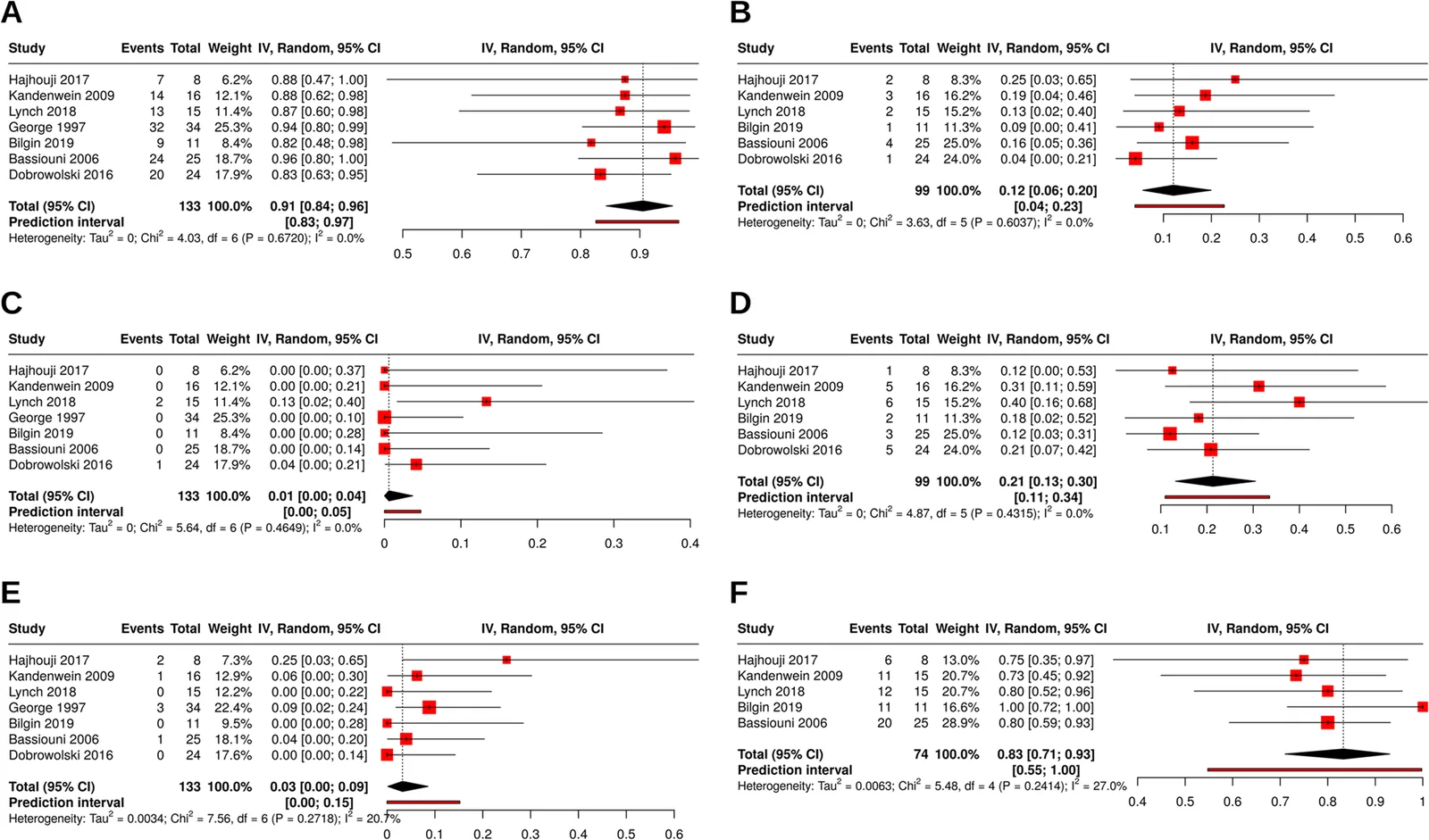
Forest plots of pooled oncologic, functional, and morbidity outcomes. (**A**) gross total resection, (**B**) CSF leak, (**C**) recurrence, (**D**) new cranial nerve deficits, (**E**) perioperative mortality, (**F**) long-term functional independence. Pooled proportions (random-effects, Freeman–Tukey transformation) with 95% confidence intervals; study-level event counts and denominators are shown for each analysis. This figure is original to this submission so no credit or license is needed

All data supporting the findings are derived from previously published studies cited in the reference list; the extracted study-level data are presented in Tables 1 and 2; Fig. 2, and additional extracted data are available from the corresponding author upon reasonable request.

**Table 1:** Study and patient characteristics

Study	Country/Center	Design	*N*	Age	Female	Tumor location	Approach	Follow-up
Hajhouji et al., 2017 [8]	Morocco – Mohammed VI University Hospital, Marrakesh	Retrospective case series	8	Mean 46.75 y (range 23–60)	62.5% (5/8)	Anterolateral 62.5% (5/8), Anterior 37.5% (3/8)	Posterior midline suboccipital + C1 laminectomy	Median 20 months (range 3–48)
Kandenwein, et al. 2009 [7]	Germany – University of Ulm	Retrospective case series	16	Mean 61 y (40–85)	75% (12/16)	Ventral (3), ventrolateral (10), dorsal (1), dorsolateral (2)	Posterior suboccipital ± lateral extension	Mean 43.5 months (24–119)
Lynch et al., 2018 [9]	Brazil – Public Servants Hospital & Rede D’or/São Luiz Hospitals, Rio de Janeiro	Retrospective case series	15	Mean 48.5 y (33–63)	80% (12/15)	80% anterior/anterolateral; 66% crossed midline	Lateral suboccipital retrosigmoid retrocondylar (condyle preserved)	Mean 10.2 years (1–24)
George et al., 1997 [3]	France – Hôpital Lariboisière, Paris	Retrospective case series (intradural subset of 40)	34	Mean 51.6 y (14–76; whole series)	76.5% (26/34)	Intradural subset: anterior 18, lateral 15, posterior 1	Posterolateral (30), posterior midline (4); 6 extradural-component cases (anterolateral approach in 5) excluded	Mean 4.8 years (1–10; whole series)
Bassiouni et al., 2006 [5]	Germany – University Hospitals Essen & Frankfurt	Retrospective case series	25	Mean 59.2 y (33–78)	76% (19/25)	Anterior 36% (9/25), Anterolateral 64% (16/25)	Posterolateral suboccipital retrocondylar (no condyle drilling; no VA transposition)	Mean 6.1 years (1–14)
Dobrowolski et al., 2016 [18]	Germany – Eberhard Karl University, Tübingen	Retrospective case series	24	Mean 52 y (10–82)	75% (18/24)	Anterior, anterolateral, and posterior (anterolateral most common)	Midline suboccipital subtonsillar + C1 posterior arch removal (posterior 1/3 condyle in 2)	Mean 45.6 months (6 mo–8 y)
Bilgin et al., 2019 [10]	Turkey – Adana City Training and Research Hospital	Retrospective case series	11	Mean 60.8 y (32–75)	72.7% (8/11)	Posterolateral 5 (46%), Anterolateral 2 (18%), Lateral 2 (18%), Anterior 2 (18%)	Posterior midline suboccipital + C1 laminoplasty (7, 64%); Far-lateral with 1/3 condyle resection (4, 36%)	Mean 18 months (12–48)

**Table 2:** Surgical outcomes and functional morbidity reported across included series

Study	Extent of resection	Recurrence	Mortality	Neuro improvement	New CN deficits	Other complications	Long-term outcome
Hajhouji et al., 2017 [8]	GTR 87.5% (7/8); STR 12.5% (1/8)	0	2/8 (25%) – postoperative pneumonia/swallowing difficulties	KPS 72.5 → 85 at last FU; 6/8 functionally independent	Transient worsening in 1 (12.5%); no persistent new CN palsy reported	CSF leak 2 (25%), meningitis 1 (12.5%), transient worsening 1 (12.5%)	6/8 alive and functionally independent
Kandenwein, et al. 2009 [7]	GTR 87.5% (14/16); STR 12.5% (2/16)	0	1/16 (6.25%) – myocardial infarction	8/16 improved ≥ 1 McCormick; 5 stable; 2 worsened; 11/15 McCormick I–II long term	Persistent IX/X/XII in 1; transient XII in 2; transient X in 1; persistent mild IX–XII paresis in 1	CSF leak 3 (1 required revision), meningitis 1, pneumonia/circulatory instability 1 (fatal)	11/15 with McCormick I–II at long-term
Lynch et al., 2018 [9]	GTR 86.6% (13/15); Simpson II (13/15), III (2/15)	2/15 (13.3%)	0 periop; 2 late deaths (13.3%) aspiration pneumonia	12/15 (80%) GOS 4–5	6/15 (40%) – IX/X (4), XII (1), VII (1); 3 partially recovered	CSF leak 2 (13.3%); temporary tracheostomy 2; venous air embolism 1; brachial plexus paresis 1	Majority (80%) functionally independent (GOS 4–5)
George et al., 1997 [3]	Intradural subset: Simpson I–II 32/34 (94.1%); Simpson III 1; Simpson IV 1	0 during follow-up	3/34 (8.8%), all perioperative – air embolism, pulmonary embolism (day 6), preoperative coma/tetraplegia (day 5)	Whole series: 90% improved, 2.5% stable, 7.5% worsened (severity index; not pooled)	Not quantified (not pooled)	Air embolism (fatal 1), pulmonary embolism (fatal 1), swallowing dysfunction in some	No validated functional scale reported (not pooled); no recurrences
Bassiouni et al., 2006 [5]	Simpson II 96% (24/25); Simpson III 4% (1/25)	0	1/25 (4%) – tracheostomy-related complication (mediastinitis)	KPS 79 → 89; 20/25 (80%) regained full daily activity	3/25 (12%) – permanent partial XII (1), transient XI (2)	CSF leak 4 (16%; 3 repaired surgically), air embolism 1, transient hemiparesis 1, extradural hematoma 1, wound infection 1, permanent tetraplegia 1 (re-do case)	80% full daily activity (KPS 90–100) at mean 6.1 y; no recurrence
Dobrowolski et al., 2016 [18]	GTR 83.3% (20/24; Simpson I 4, II 15, III 1); Simpson IV 4 (16.7%)	1/24 (4.2%) – regrowth after subtotal removal	0	All patients improved in motor power; sensory deficits resolved in 5, improved in 3	5/24 (20.8%) – all transient: new XII paresis (1), dysphagia (1), dysarthria + dysphagia (1); transient worsening of pre-existing lower CN deficits (2)	Postoperative hematoma requiring evacuation, then CSF leak + hydrocephalus (VP shunt) 1; pneumonia + transverse sinus thrombosis 1	No recurrence among totally removed tumors at mean 45.6 months
Bilgin et al., 2019 [10]	GTR 82% (9/11); STR 18% (2/11)	0	0	KPS 70 → 80; VAS for neck pain decreased in posterior/posterolateral cases	2/11 (18%) temporary IX/X palsy (far-lateral cases)	CSF leak 1 (9%) managed with lumbar drainage; persistent neck pain in some condyle resection cases	11/11 with postoperative KPS ≥ 80 (80–90) at follow-up

## Results

### Study identification

Seven retrospective case series (Table 1) published between 1997 and 2019 met the inclusion criteria, collectively contributing 133 eligible patients with FMMs managed via posterior midline suboccipital and/or lateral/posterolateral suboccipital (retrosigmoid/retrocondylar) corridors. For George et al. [3], the analysis was restricted to the 34 intradural cases operated through posterolateral (*n* = 30) or midline posterior (*n* = 4) approaches, in which, as stated in the primary report, the vertebral artery was never transposed and no more than one-third of the condyle or lateral mass of the atlas was drilled; the 6 cases with an extradural component, 5 of which were treated through an anterolateral approach, were excluded, and outcomes for the retained subset were extracted from the patient-level table of the primary report. Many published FMM series were excluded because outcomes for these corridors could not be separated from more extensive transcondylar/far-lateral or other skull-base approaches, or because cohorts were too small (< 5) or lacked extractable endpoints. Notable full-text exclusions included the multi-institutional series of Fatima et al. [13] and Gattozzi et al. [14], in which far-lateral/extreme-lateral or transcondylar outcomes could not be separated from the corridors of interest; the series of Giordano et al. [15], in which anterior lesions were managed with dorsolateral transcondylar-family exposures without corridor-specific outcome stratification; and the series of Pirotte et al. [16], in which the vertebral artery was transposed in 19 of 22 operated patients and the occipital condyle drilled in 10. In addition, the earlier report by Lynch et al. (2013) [17] was excluded as an overlapping cohort of the same institutions’ subsequent series (recruitment 1986–2011 contained within 1986–2016 [9]), retaining the most complete report in accordance with our prespecified criteria. All 31 reports previously excluded at full text as using an ineligible approach were re-screened during revision; apart from Bassiouni et al. [5] and Dobrowolski et al. [18], none met the eligibility criteria. Accordingly, the included studies were either series dedicated to a single corridor of interest or mixed-approach cohorts in which outcomes attributable to the posterior/posterolateral corridors were clearly reportable. The studies originated from France (*n* = 34 eligible of 40) [3], Germany (*n* = 16, *n* = 25, and *n* = 24) [5, 7, 18], Brazil (*n* = 15) [9], Morocco (*n* = 8) [8], and Turkey (*n* = 11) [10]. Several included cohorts used a mix of midline and lateral/posterolateral corridors with the approach chosen by tumor attachment [3, 7, 10], whereas the Lynch et al. (2018) and Bassiouni et al. series used a dedicated lateral suboccipital retrosigmoid/retrocondylar corridor and the Hajhouji et al. and Dobrowolski et al. series a dedicated posterior midline suboccipital corridor [5, 8, 9, 18]. The overall cohort was predominantly female (100/133 patients; 75.2%).

### Outcomes

The pooled weighted mean age at presentation was 54.4 years, with individual study means ranging from 46.75 years [8] to 61 years [7]. The sample-size-weighted mean follow-up across the included studies was 58 months, with study-level central values ranging from 18 months to a mean of 10.2 years; one study reported median rather than mean follow-up, a heterogeneity acknowledged in the Limitations. The most frequently reported presenting symptoms were cervico-occipital pain and posterior headache, documented universally in 2 series and in 58–87% of patients across the remainder. Motor deficits including pyramidal syndrome, hemiparesis, and quadriparesis were the second most prevalent complaint, affecting 32–100% of patients depending on the series. Gait disturbance or ataxia was present in 21–50% of patients, while LCN dysfunction, particularly dysphagia reflecting involvement of CN IX and X, was documented in 8–54% of patients. George et al. [3], notably included a proportion of severely compromised patients, with tetraplegia and hemiplegia in 25% and 12.5% of their cohort, respectively. Tumor attachment (Table 1) was predominantly anterior/anterolateral or ventrolateral across the included series. Resections were performed via posterior midline suboccipital corridors (often with C1 laminectomy/laminoplasty) and/or lateral/posterolateral suboccipital retrosigmoid/retrocondylar corridors, with approach selection typically tailored to tumor position [3, 7, 9], while the Lynch and Bassiouni series focused on the retrosigmoid/retrocondylar route and the Dobrowolski series on the midline subtonsillar route [5, 18]. Occipital condyle work was generally minimized: most series reported no condylectomy, and when performed it was limited to ≤ 1/3 of the condyle (e.g., 3/16 in Kandenwein et al. [7], 2/24 in Dobrowolski et al. [18], subset in Bilgin et al. [10]), with no reported postoperative craniocervical instability or need for fusion.

The pooled GTR rate (Table 2; Fig. 2A) across all 7 studies was 91% (95% CI: 84–96%; 119/133 patients), confirming that posterior and posterolateral approaches are oncologically effective for the majority of FMMs [3, 5, 7–10, 18]. This high resection rate translated into durable tumor control: recurrence or regrowth was recorded in 3/133 patients (2.3%; two in Lynch et al. [9] and one after subtotal removal in Dobrowolski et al. [18]), corresponding to a pooled estimate of 1% (95% CI: 0–4%; 7 studies; Fig. 2C). Because pooled proportions are unstable for such rare events, the crude proportion is reported alongside the pooled value. Adjuvant treatment was rarely required; only the Lynch et al. [9] series reported radiotherapy for recurrence during long-term follow-up (one patient), while all other series reported no adjuvant intervention. Follow-up duration ranged from a median of 20 months to a mean of 10.2 years [8, 9], with the Brazilian series providing follow-up extending beyond two decades in selected patients and the Bassiouni et al. series contributing a mean of 6.1 years, together offering the most robust long-term oncological data in the cohort [5, 9].

Functional and neurological outcomes (Table 2) were favorable across the included series [3, 5, 7–9, 18]. Among the five series reporting a validated functional scale, 83% of patients (95% CI: 71–93%; 60/74 patients; I² = 27%) were functionally independent at long-term follow-up (Fig. 2F). Neurological improvement relative to the preoperative status was reported by most series, but on the same scales as functional independence, and was therefore not pooled as a separate outcome. Outcome was assessed using heterogeneous scales: GOS 4–5 was achieved in 80% of patients in the Lynch series [9], McCormick grade I–II in 73.3% of evaluable patients in Kandenwein et al. [7], and mean KPS improved from 72.5 to 85 in Hajhouji et al. [8] and from 79 to 89 in Bassiouni et al., with 80% of the latter cohort regaining full daily activity [5], and all 11 patients in Bilgin et al. had a Karnofsky score of 80–90 at follow-up [10]. George et al. [3] reported clinical improvement in 90% of the whole series on an unvalidated severity index, and all patients in Dobrowolski et al. improved in motor power [18]; neither measure met the definition of functional independence, and neither was pooled.

Regarding surgical morbidity, new CN deficits (transient or permanent) were identified in 21% of patients (95% CI: 13–30%; 22/99 patients, 6 studies; I² = 0%; Fig. 2D), and CSF leakage occurred in 12% (95% CI: 6–20%; 13/99 patients, 6 studies; Fig. 2B) [5, 7–10, 18]. George et al. [3] did not quantify lower cranial nerve palsies or CSF leak and could not contribute to these estimates; in Kandenwein et al., five patients developed new cranial nerve deficits, four of them transient or mild [7]; in Dobrowolski et al., the patient-level table records a postoperative cranial nerve deficit in five patients, all transient (new hypoglossal paresis, dysphagia, and dysarthria with dysphagia in one patient each, and transient worsening of pre-existing lower cranial nerve deficits in two) [18]. Additional complications included meningitis in 2 series, aspiration pneumonia necessitating temporary tracheostomy in 2 patients in Lynch et al. [9], and isolated cases of brachial plexus paresis and venous air embolism. In Bassiouni et al., one extradural hematoma required evacuation and one previously irradiated re-do case remained tetraplegic [5], while one patient in Dobrowolski et al. required hematoma evacuation followed by ventriculoperitoneal shunting for hydrocephalus [18]. Persistent neck pain was observed in a subset of patients who underwent condylectomy in Bilgin et al. [10], underscoring the potential functional cost of more extensive bone work. Two late deaths attributable to aspiration pneumonia from persistent lower CN dysfunction were recorded during follow-up in the Lynch et al. series [9], illustrating a distinct delayed morbidity profile of FMMs; these deaths occurred beyond the perioperative period and were not counted as perioperative mortality. One perioperative tracheostomy-related death occurred in Bassiouni et al. [5]. The pooled perioperative mortality rate was 3% (95% CI: 0–9%; 7/133 patients, 7 studies; I² = 21%), with moderate imprecision reflecting the small total sample size (Fig. 2E); including the two late deaths, all-cause mortality during follow-up was 9/133 (6.8%) [3, 5, 7–10, 18].

### Outcomes by surgical corridor

Because the two corridors are anatomically distinct and were applied to topographically different tumors, outcomes were additionally summarized descriptively by approach. In approach-pure retrosigmoid/retrocondylar series (Lynch et al. and Bassiouni et al. [5, 9]; *n* = 40, predominantly anterior/anterolateral attachments), GTR was achieved in 37/40 patients (92.5%), new cranial nerve deficits occurred in 9/40 (22.5%), CSF leak in 6/40 (15.0%), perioperative mortality in 1/40 (2.5%) with two further late aspiration-related deaths (5.0%), and recurrence in 2/40 (5.0%). In approach-pure midline suboccipital series (Hajhouji et al. and Dobrowolski et al. [8, 18]; *n* = 32, mixed attachments including anterior/anterolateral lesions), GTR was achieved in 27/32 patients (84.4%), new cranial nerve deficits in 6/32 (18.8%, all transient), CSF leak in 3/32 (9.4%), perioperative mortality in 2/32 (6.3%), and recurrence in 1/32 (3.1%). The remaining mixed-approach cohorts (Kandenwein et al., the eligible George et al. subset, and Bilgin et al. [3, 7, 10]; *n* = 61) selected the corridor according to tumor attachment and did not report outcomes separately by approach; in the George et al. subset, posterolateral (*n* = 30) and midline posterior (*n* = 4) exposures are combined within a single dataset [3]. Because approach selection was guided by tumor topography in all series, these strata are descriptive and must not be interpreted as a comparative analysis.

**Fig. 3:**
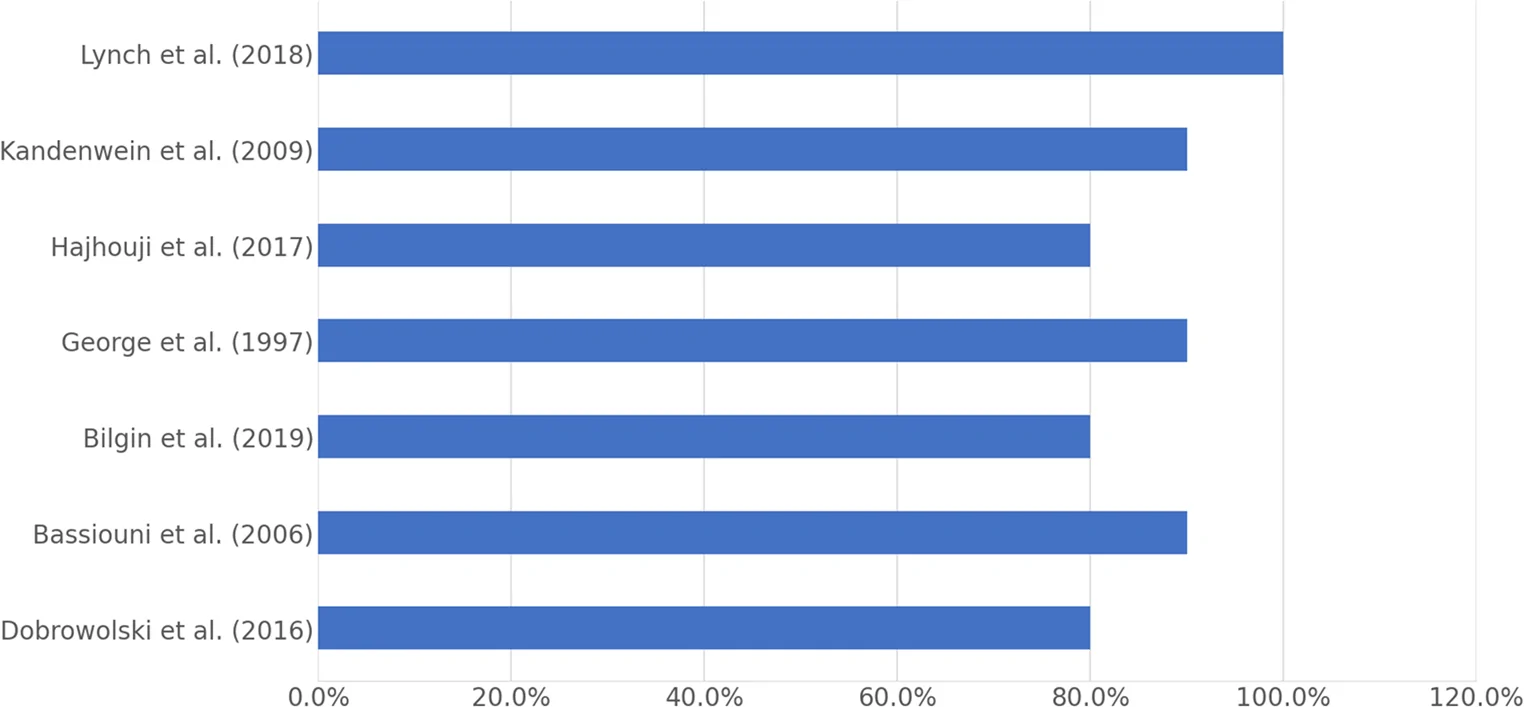
Methodological quality (risk of bias) assessment of included case series using the Joanna Briggs Institute (JBI) critical appraisal tool (bar chart of percent scores). Percent scores represent the proportion of applicable JBI criteria met; 100% indicates the highest methodological quality. Studies are labeled by first author and publication year. This figure is original to this submission so no credit or license is needed

Methodological quality of the included series was moderate to high (Fig. 3): JBI percent scores were 100% for Lynch et al. [9]; 90% for Kandenwein et al. [7], George et al. [3], and Bassiouni et al. [5]; and 80% for Hajhouji et al. [8], Bilgin et al. [10], and Dobrowolski et al. [18].

## Discussion

In this single-arm meta-analysis of retrosigmoid/retrocondylar and midline suboccipital approaches for FMMs, we found that contemporary posterior and posterolateral corridors achieve high extents of resection with acceptable rates of CSF leak; however, functional morbidity is primarily driven by LCN deficits. Pooled estimates indicated a GTR rate of 91% (95% CI: 84–96%), a new postoperative cranial nerve deficit rate of 21% (95% CI: 13–30%), a postoperative CSF leak rate of 12% (95% CI: 6–20%), and perioperative mortality of 3% (95% CI: 0–9%), concordant with the values reported in the Abstract and Results. These results emphasize that the central trade-off in FMM surgery is not merely “more exposure versus less exposure,” but rather “maximal safe resection versus preservation of brainstem and LCN function,” particularly in ventrolateral disease.

### Retrosigmoid/retrocondylar corridor

When examined by corridor, the approach-pure retrosigmoid/retrocondylar series [5, 9], composed almost entirely of anterior and anterolateral tumors, achieved GTR in 92.5% of patients (37/40) without condylar resection or vertebral artery transposition, at the cost of new, predominantly transient, cranial nerve deficits in 22.5%, perioperative mortality of 2.5%, and two late aspiration-related deaths (5.0%) during follow-up. These figures support the contemporary consensus that a well-executed retrocondylar exposure suffices for the vast majority of ventral and ventrolateral FMMs: the tumor itself progressively creates the working corridor as it is debulked, condylar drilling can be limited to a tailored posterior-third resection when additional exposure is genuinely required, and a truly extensive transcondylar approach is almost never necessary [5, 9, 19–21].

### Midline suboccipital corridor

The approach-pure midline suboccipital series [8, 18] achieved GTR in 84.4% of patients (27/32) with new cranial nerve deficits in 18.8% (6/32; all transient) and CSF leak in 9.4%. It must be emphasized, however, that the midline suboccipital and retrocondylar corridors are not interchangeable operations. The midline route provides direct access to posterior and posterolateral tumors and, in the subtonsillar variant, has been extended to selected anterior and anterolateral lesions by highly experienced teams [18]; it should not, on the basis of the present pooled data, be regarded as an equivalent alternative to a far-lateral/retrocondylar exposure for ventral disease. Because approach selection in every included series was dictated by tumor attachment, the favorable pooled estimates reflect corridors applied to the lesions they suit best, a form of confounding by indication. Trainees in particular should note that the far-lateral/retrocondylar approach remains the workhorse for anterior and anterolateral FMMs, with the midline corridor reserved for posterior/posterolateral lesions and carefully selected exceptions [2, 5, 6, 18].

### Comparison with transcondylar and far-lateral approaches

Our pooled estimates also compare favorably with published outcomes of transcondylar and far-lateral strategies. In the series of Pirotte et al., in which the vertebral artery was transposed in 19 of 22 operated patients and the condyle drilled in 10, GTR was achieved in 73% with one death and four permanent deficits [16]. Large contemporary cohorts employing tailored condylar resection similarly report GTR rates in the range of 70–90%, with functional outcome governed chiefly by vertebral artery encasement, tumor size, and anatomic subtype rather than by the extent of bone removal [22, 23]. A prior meta-analysis encompassing all surgical approaches reported an overall complication rate of 17.2% and recurrence of 2.5% [4]. Against this background, the pooled GTR of 91%, cranial nerve morbidity of 21%, and perioperative mortality of 3% achieved through condyle-sparing corridors suggest that, for appropriately selected tumors, limited-exposure strategies do not compromise oncologic or functional outcomes; this comparison is nonetheless indirect, as transcondylar series often include larger, more ventral, or artery-encasing lesions [4, 16, 23].

Our pooled outcomes align with major institutional series and reviews indicating that most FMMs can be resected with acceptable mortality when the surgical approach is tailored to tumor anatomy [2–4]. For instance, high Simpson-grade resections with low mortality have been achieved using retrocondylar and posterolateral routes [5], supporting the notion that adequate exposure of the dural base and neurovascular interfaces, rather than routine maximal bone removal, often determines the feasibility of safe gross total resection.

### Approach selection and classification

Approach selection is optimally framed using “surgical corridor” and classification principles that integrate: (1) dural attachment relative to the dentate ligament, (2) relationship to the vertebral artery (VA), and (3) laterality or extension across the midline [1, 2, 6]. Posterior midline suboccipital approaches remain appropriate for posterior and posterolateral tumors, whereas retrosigmoid-lateral suboccipital retrocondylar modifications expand the working angle for lateral and selected anterolateral tumors without necessarily requiring condylar resection [7, 9]. These paradigms correspond with broader skull base literature demonstrating that modern “far-lateral family” approaches evolved from lateral suboccipital concepts to access ventrolateral craniovertebral pathology while minimizing brainstem traction [22, 24, 25].

### Occipital condyle management

A key technical controversy concerns the necessity of routine occipital condyle drilling. Multiple clinical series indicate that most FMMs can be removed via retrocondylar strategies without condylectomy when meticulous arachnoid dissection and VA and LCN control are achieved [5, 9]. Quantitative and anatomic studies demonstrate that incremental condylar resection improves exposure and maneuverability but must be balanced against the risk of craniocervical instability, underscoring a “minimal bone, maximal corridor” philosophy [19–21]. Practically, condyle drilling should be individualized and reserved for lesions with limited lateral working angles, VA encasement, or when safe access to the dural base is otherwise unattainable.

### Cranial nerve morbidity

Despite low pooled CSF leak and mortality rates, the predominant functional morbidity was postoperative cranial nerve dysfunction. This finding is biologically plausible given the high density of LCNs and the perforator-rich vertebral artery/posterior inferior cerebellar artery (VA/PICA) region at the foramen magnum. Classification systems incorporating VA–tumor relationships can predict LCN displacement patterns (e.g., superior displacement when tumors grow below the VA), potentially facilitating safer dissection strategies and neuromonitoring planning [6]. Larger contemporary cohorts further demonstrate that VA encasement, extradural extension, and complex anatomic subtypes predict poorer functional outcomes and increased progression or recurrence risks, supporting risk-stratified surgical goals [23].

### Alternative anterior corridors

Alternative anterior routes, such as transoral/transpharyngeal or endoscopic endonasal approaches, provide direct access to midline ventral pathology but are limited by narrow corridors, restricted lateral reach, and concerns regarding reconstruction and CSF leaks [2, 26, 27]. Recent anatomical comparisons suggest that endonasal “far-medial” approaches may complement, rather than replace, posterolateral routes, emphasizing that approach selection should be lesion-specific and dependent on center expertise [28, 29]. When gross total resection is unsafe due to adherence to critical neurovascular structures, planned subtotal resection combined with adjuvant radiosurgery or observation may be appropriate [4, 16].

### Limitations

This study has limitations common to surgical meta-analyses, including predominantly retrospective series, heterogeneity in tumor subtype distributions, variable definitions of “cranial nerve deficit” and “complications,” and inconsistent reporting of functional scales and follow-up imaging. Additionally, the small number of included studies (k = 7) precluded sensitivity analyses, formal heterogeneity exploration, and valid funnel plot assessment; the absence of GRADE ratings reflects the uniformly low evidence certainty of the included series rather than an oversight in review methodology. Additional limitations warrant emphasis: the two corridors, although both condyle-sparing, are anatomically and technically distinct operations that were pooled in a single-arm design; because approach selection was guided by tumor attachment in every included series, the pooled estimates are subject to confounding by indication, and the corridor-stratified figures presented in the Results are descriptive only. The mixed-approach cohorts (Kandenwein et al., Bilgin et al., and the eligible George et al. subset [3, 7, 10]) could not be decomposed by corridor, so the number of patients treated by a purely midline exposure remains modest. Denominators differ across outcomes: cranial nerve deficits and CSF leak could be pooled from only 99 patients and functional independence from 74, because George et al. [3], the largest series, did not quantify lower cranial nerve palsies or CSF leak and reported no validated functional scale; as that series recorded three perioperative deaths and unquantified lower cranial nerve palsies, the pooled morbidity estimates may be biased downward. The review was not prospectively registered, no protocol was prepared, and the full database search strings are not reproduced verbatim. Finally, follow-up summaries combined study-level means with medians, and the weighted mean follow-up should be interpreted with corresponding caution. Future research should standardize reporting, such as LCN outcomes by individual nerve, temporary versus permanent deficits, VA encasement, Simpson grade, and patient-reported quality of life, and analyze outcomes using validated anatomic classifications to better personalize surgical corridor selection and predict morbidity.

## Conclusion

Posterior and posterolateral surgical corridors, including the retrosigmoid/retrocondylar and midline suboccipital approaches, can achieve effective tumor control for foramen magnum meningiomas while preserving functional status in most patients when the surgical corridor is matched to tumor topography. The favorable pooled estimates reported here represent a combined figure across two surgically distinct strategies applied to different lesions (retrosigmoid/retrocondylar exposure predominantly for anterior/anterolateral tumors and midline suboccipital exposure predominantly for posterior/posterolateral tumors), and should not be interpreted as establishing the equivalence or interchangeability of the two approaches. Within this framework, these corridors frequently enable safe resection without the need for routine extensive condylar drilling, aligning with the principle of maximal safe resection. Nevertheless, clinically significant morbidity remains, particularly lower cranial nerve dysfunction and cerebrospinal fluid-related complications, highlighting the importance of meticulous surgical technique and proactive perioperative airway and swallowing management. Future multicenter studies with standardized, approach-stratified outcome reporting are essential to refine corridor selection and enhance patient counseling.

## Data Availability

All data supporting the findings are derived from previously published studies cited in the reference list; the extracted study-level data are presented in Tables 1 and 2; Fig. 2, and additional extracted data are available from the corresponding author upon reasonable request.

## Declarations

### Registration and Protocol

This systematic review was not registered in PROSPERO or any other registry, and no review protocol was prepared prior to its conduct.

### Previous Presentation

This work was presented as a podium presentation at the North American Skull Base Society (NASBS) Annual Meeting, San Diego, California, USA, March 2026 (Gupta K, Karsy M. Surgical Outcomes and Functional Morbidity Following Retrosigmoid and Midline-Suboccipital Approaches for Foramen Magnum Meningiomas: A Single-Arm Meta-Analysis).
